# Pressure Dependent Changes in CO_2_‐Concentrations Modulate the 3‐Hydroxyvalerate Fraction in Terpolymeric Polyhydroxyalkanoates

**DOI:** 10.1002/biot.70298

**Published:** 2026-08-20

**Authors:** Birk Achenbach, Pravesh Tamang, Günter E. M. Tovar, Susanne Zibek

**Affiliations:** ^1^ Institute of Interfacial Process Engineering and Plasma Technology IGVP University of Stuttgart Stuttgart Germany; ^2^ Fraunhofer Institute for Interfacial Engineering and Biotechnology IGB Stuttgart Germany

**Keywords:** carbon dioxide, Cupriavidus necator, heterotrophic growth, PHBVV, polyhydroxyalkanoate

## Abstract

Polyhydroxyalkanoates (PHAs), especially their copolymeric forms, such as poly(3‐hydroxybutyrate‐co‐3‐hydroxyvalerate‐co‐4‐hydroxyvalerate) (PHBVV), have promising properties for replacing petrochemical‐derived plastics. However, the research focuses mostly on producing PHA from a specific substrate or waste‐stream, while the reproducibility of a precise polymer composition is seldom targeted. This study systematically focuses on the influence of pressure conditions on the production of terpolymeric PHBVV by *Cupriavidus necator* NCIMB 11599 using levulinic acid (LA). Three different process setups were evaluated to assess the impact of hydrostatic pressure (Δ*p*_Hydro_) and applied pressure (Δ*p*_Vessel_) compared to the process at ambient pressure. Differences were detected regarding carbon utilization (Y_X/Gluc_ & Y_PHA/LA_) and polymer composition. The Y_X/Gluc_ increased in the pilot scale compared to the technical scale from 0.31 ± 0.01 g_X_ g_Gluc_^−1^ to 0.41 ± 0.01 g_X_ g_Gluc_^−1^. Conversely, as Δ*p*_Vessel_ increased, the Y_PHA/LA_ decreased from 0.35 ± 0.02 g_PHA_ g_LA_^−1^ to 0.23 ± 0.02 g_PHA_ g_LA_^−1^, while the corresponding 3HV contents increased from 48 ± 1%_(w/w)_ to 69 ± 2%_(w/w)_. These changes were consistent with pressure‐dependent variations in CO_2_ concentration and a potentially increased carbon fixation via the energy‐intensive CBB cycle. This indicates that the polymer composition and cell vitality can be adjusted via the CO_2_ concentration, a factor that should be taken into account when further scaling up of PHA production processes.

## Graphical Abstract and Lay Summary

The influence of pressure conditions on the production and composition of terpolymeric PHA were investigated in the technical and the pilot scale. The impact on Y_X/LA_ & Y_PHA/LA_ and polymer composition (Y_X/Gluc_: 0.31 ± 0.01 g_X_ g_Gluc_^−1^→0.41 ± 0.01 g_X_ g_Gluc_^−1^; Y_PHA/LA_: 0.35 ± 0.02 g_PHA_ g_LA_^−1^→0.23 ± 0.02 g_PHA_ g_LA_^−1^; 3HV content: 48 ± 1%→69% ± 2%) has been connected to the variation in CO_2_ concentration resulting from the different pressure conditions, highlighting an important key parameter for consideration during scaleup.

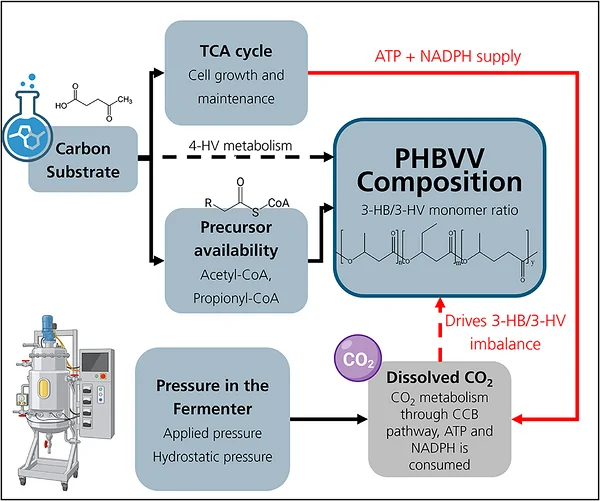

## 1 Introduction

**FIGURE 1:**
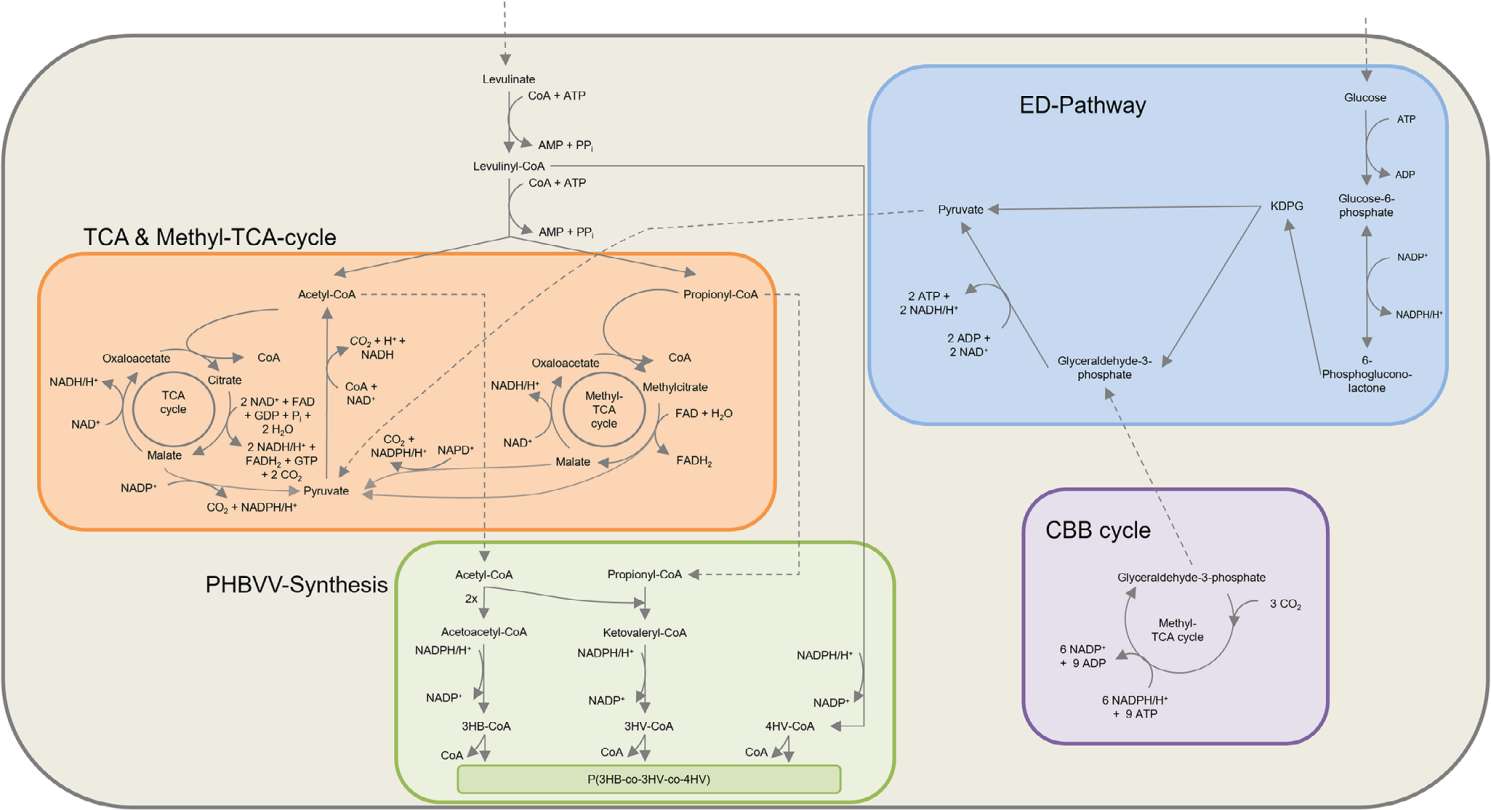
Metabolic pathways in *C. necator* covering catabolism from glucose and levulinic acid [4, 5, 17] and PHBVV synthesis from levulinic acid [4, 5]; CO_2_ is produced in the TCA cycle [17] and during the decarboxylation of malate [15] and pyruvate and reutilized in the CBB cycle [10, 14, 17]; provision of reduction equivalents and ATP by the TCA‐cycle with adjacent reactions and the ED‐Pathway [15, 17], as well as the Methyl‐TCA cycle [18, 19], adapted from [20].

Polyhydroxyalkanoates (PHA) are a group of biopolyesters produced by various microorganisms. Their production is a cell response to carbon abundance paired with a growth limitation, such as a nutrient deficiency or cellular stress [1]. When growing chemolithoautotrophically with CO_2_/H_2_ [2] or heterotrophically with structurally unrelated substrates, such as glucose, the model organism *Cupriavidus necator* produces the homopolymer polyhydroxybutyrate (PHB) [3]. However, if specific structurally related organic precursors, such as levulinic acid, are supplied in the medium, it leads to the production of a terpolymeric poly(3‐hydroxybutyrate‐co‐3‐hydroxyvalerate‐co‐4‐hydroxyvalerate) (PHBVV) (Figure 1) [4, 5]. This terpolymer is of special interest, since the two species of hydroxyvalerate enable a greater opportunity to influence the properties of the material than the commonly investigated copolymer PHBV containing only 3‐hydroxyacids [6, 7, 8, 9].

*C. necator* is not only capable of using organic precursors in combination with supplied CO_2_ in mixotrophic growth, but can also reutilize autogenous CO_2_, which originates from the cellular metabolism itself during heterotrophic growth [10, 11]. *C. necator* generally preferentially utilizes organic carbon over the carbon dioxide during growth, showing diauxic or mixotrophic behavior depending on the overall carbon concentration. Interestingly, during PHA production, CO_2_ appears to be preferentially assimilated compared to other carbon sources [12].

Both mixotrophic and heterotrophic usage of CO_2_ increases the biomass yield Y_X/S_ compared to the usage of only organic carbon [10, 13]. If the CO_2_ supply availability becomes excessive, the effect of enhanced yield is overshadowed by an inhibitory character of CO_2_, which leads to decreased final biomass and product titer. This was reported in lab scale during mixotrophic growth [12] and during the scale‐up of a heterotrophic production process to the 300‐L‐scale [3]. This can be explained by the role of the Calvin‐Benson‐Bassham (CBB) cycle for CO_2_ fixation.

The enzymes of the Calvin‐Benson‐Bassham (CBB) cycle for CO_2_ fixation are highly expressed even during heterotrophic growth [10, 11]. This puts large parts of the proteome on standby, which places a burden on the cellular catabolism. Grunwald et al. [14] showed that fixation of one mole of CO_2_ via the CBB cycle requires the equivalent of three moles of NADH. During heterotrophic growth, this demand in reduction equivalents is likely to be fueled by the TCA cycle and the oxidative carboxylation of malate [15, 16], which reduces availability for other parts of the metabolism.

While with homopolymeric PHB the overall titer is reduced by this catabolic burden, there might be an even greater effect with copolymeric PHBVV. The ratio of produced 3HB and 3HV monomers is directly dependent on the available pool of acetyl‐CoA and propionyl‐CoA (Figure 1). We assume that this balance is shifted by an increased activity of the CBB‐cycle, since more acetyl‐CoA gets funneled into the TCA cycle as a main source for the reduction equivalents needed for the CO_2_ fixation. This would shift the available pool of acetyl‐CoA and propionyl‐CoA for PHBVV synthesis, which would increase the 3‐hydroxyvalerate (3HV) fraction of the polymer. Since 4‐hydroxyvalerate (4HV) formation is independent of the ratio of acetyl‐CoA and propionyl‐CoA, it should not be influenced by varying CO_2_ availability.

In previous studies regarding the role of CO_2_ on PHB production, the influence was investigated by altering the CO_2_ content in the aeration from 0.04% to 22% and the rate of aeration from 0.4 to 1.0 vvm [14]. Particularly during scale‐up of the process, CO_2_ becomes a critical factor. On one hand, increased applied pressure is often used in stainless steel vessels to prevent contamination from the outside. On the other hand, water columns quickly reach several meters in industrial scale bioreactors. In a typical 10 m^3^ scale bioreactor with an H/D of three and a working volume of 50%, the water column would already reach 2.4 m exercising a hydrostatic pressure of 0.24 bar.

The objective of this study was to elucidate the relationship between total pressure (applied and hydrostatic) in the fermenter, CO_2_ solubility, and PHA composition. Specifically, the work aimed to determine how pressure‐dependent changes in dissolved CO_2_ concentrations modulate metabolic fluxes within the PHA pathway and how these effects translate into variations in PHBVV copolymer composition.

## 2 Materials and Methods

### 2.1 Fermentation

The strain used was *C. necator* NCIMB 11599, maintained at −80°C in LB medium containing 16% glycerol. Prior to each fermentation, the cryo‐culture was reactivated by streaking onto an LB agar plate (15 g L^−1^ agar) and incubated for >48 h at 30°C.

For preculture step 1 (PC 1), cells were resuspended in 1–2 mL LB medium before transferring into 100–200 mL of LB medium in 20% filled shake flasks. It was incubated until the broth reached an OD > 0.3 (600 nm). For PC 2, 10% of PC1 was used to inoculate the MS medium according to Achenbach et al. [20] and incubated for 16–18 h before inoculating into the production bioreactor. All shake flask cultures were incubated at 30°C and 120 rpm. For the pilot scale, several PC1 flasks were pooled and used for inoculation of the PC2, which was cultivated in a 100‐L seed reactor with 55 L working volume for 15 h.

The fermentation was performed in two different scales: the technical scale in a 42‐L‐bioreactor (Bioengineering, Switzerland) and the pilot scale in a 1000‐L‐bioreactor (Heinrich Frings GmbH & CO. KG, Germany). The bioreactors’ specific data and operation conditions are listed in Table 1. The initial specific power intake was kept within the range of 0.14–0.16 kW m^−3^, to provide process comparability. To achieve that, the aeration had to be reduced by a factor of 10 in the pilot scale, since the specific power input solely by aeration with 1 vvm (0.17 kW m^−3^) would have exceeded the set limit. Unless otherwise stated, each fermentation condition represents one independent cultivation experiment. The cultivation with decreased pressure was already extensively characterized in Achenbach et al. [20]. The pilot scale fermentation was once repeated utilizing a modified preculture cascade. Due to significant difference in growth behavior only the replicate with a cascade comparable to the technical scale was considered for comparison although the final polymer composition did not significantly differ between both pilot scale experiments.

**TABLE 1:** Technical data of the bioreactors and growth parameters.

Parameter	Technical		Pilot
Vessel volume/L	42		1000
Initial volume/L	23		530
Reactor diameter D/m	0.3		0.8
Aeration rate/vvm	1		0.1
Initial stirrer frequency/min^−1^	219		71
Specific power input by aeration/kW m^−3^	0.05		0.02
Initial specific power input by stirring/kW m^−3^	0.11		0.12
Initial total specific power input/kW m^−3^	0.16		0.14
Applied pressure Δp_Vessel_/bar	0.125^a^	0.500^b^	0.500
Hydrostatic pressure Δp_Hydro_/bar	0.032		0.103

^a^Decreased pressure (DP) and ^b^increased pressure (IP).

As the primary carbon source during the biomass growth, glucose was used. Following glucose depletion, levulinic acid (LA) was introduced as a carbon source. LA served as the substrate for cell growth on the residual nitrogen as well as PHBVV production. It was added by a pH‐ and pO_2_‐dependent feeding strategy according to Achenbach et al. [20]. The strategy utilized two separate feed tanks containing feed 1 (50% (w/v) LA, neutralized with NaOH to pH 7) and feed 2 (70% (w/v) LA at pH 1.8). Feed 1 was controlled by the pO_2_ value and added pulse‐wise once a rising pO_2_ indicated substrate depletion, while feed 2 was controlled by the pH and therefore, continuously added. The minimum setpoint for the partial pressure of oxygen in the broth (pO_2_) was set at 20% and controlled using fermenter‐specific control software. The pH was maintained at 7.0 and regulated using 4 M KOH and feed 2. The temperature was set to 30°C. As an antifoam agent, 10% and 2% polypropylene glycol (P2000) was used for the technical and pilot scales, respectively.

### 2.2 Dissolved CO_2_ Assessment

To assess whether the expected dissolved CO_2_ concentration during fermentation is within the range suitable for utilization by *C. necator*, a preliminary experiment in the technical scale fermenter was performed. Dissolved CO_2_ was measured using the CO_2_NTROL sensor (Hamilton AG, Bonaduz, Switzerland). During the evaluation, glucose‐ and cell‐free medium were used to keep the influence of soluble ions on the CO_2_‐solubility constant. To keep the influence of the carbonic acid buffer constant, the pH was controlled at 7 with 4 M KOH.

To simulate autogenous production of CO_2_ in cell free media, respiration was substituted by the addition of 2% synthetic CO_2_ to the gas flow. This value corresponds to the exhaust gas concentration of CO_2_ with an active biomass between 8–13 g L^−1^ before the onset of PHA production known from prior fermentation experiments [20]. The gas was measured via a BlueVary analyzer (BlueSens gas sensor GmbH, Germany).

The assessment was conducted at varying applied pressures Δ*p*_Vessel,_ with 15–25 L media volume to achieve hydrostatic pressures Δ*p*_Hydro_ ranging from 0.021 to 0.034 bars. Each variation was observed until the CO_2_ level was stable for 400 s. The plateau values were averaged and plotted against used Δ*p*_Vessel_ and Δ*p*_Hydro_ resulting from the different liquid levels in the vessel to assess the influences of both parameters. The data was used for further assessment as described in Section 2.4.

### 2.3 Analytics

The samples were drawn aseptically from the bioreactors and stored on ice until further treatment. The biomass was determined as a cell dry weight (CDW), by centrifuging the samples in pre‐dried and weighed tubes. The supernatant was used for analyzing carbon and nitrogen, while the cell pellet was washed once with an equal volume of distilled water and dried at >60°C until constant weight was achieved. After determining CDW, the biomass was used for estimating PHA content and composition.

The samples for PHA analysis were prepared according to Tamang et al. [21] and measured using GC‐2010 pro (Shimadzu, Germany) equipped with a Flame ionization detector and SH‐Polarwax column (L = 30 m, ID = 0.25 mm) according to Achenbach et al. [20].

For NH_4_^+^ quantification, enzymatic ammonia assay kits (Megazyme Ltd., Wicklow, Ireland/ 4BioCell GmbH & Co. KG, Bielefeld, Germany) were used.

Levulinic acid was quantified by high‐performance liquid chromatography (HPLC). External standards from 0.3125–10 g L^−1^ were used to calibrate the system. The instrument (Shimadzu Deutschland GmbH, Duisburg, Germany/ BISCHOFF Analysentechnik u. ‐geräte GmbH, Leonberg, Germany) was equipped with a Rezex ROA‐Organic Acid H+ (8%) Ion Exclusion column (30 cm × 7.8 mm, Phenomenex, Aschaffenburg, Germany) and a refractive index detector. The mobile phase was 5 mM H_2_SO_4_ at 0.6 mL min^−1^ with the column oven set at 30°C.

Glucose was quantified by the use of enzymatic assays (R‐Biopharm, Darmstadt, Germany/ 4BioCell GmbH & Co. KG, Bielefeld, Germany) and HPLC [22].

### 2.4 Calculations

Yield coefficients were derived by regression fitting in the linear region of the respective plots. For µ_max_ the general law for exponential growth was linearized via the natural logarithm. Respective Δ*t* are given in the Supporting Information (appendix 1).

Calculation of the specific power input was done in accordance with our previous study [20].

The hydrostatic pressure *p*_hydro_ was calculated based on the gravitational force *g* and the density of water *ρ*, as well as the bioreactor diameter *D*_Vessel_ and the liquid volume *V*_L_ to obtain the height of the water column *h*_L_ under the assumption that the space taken up by the fittings is negligible (1). For simulated reactor sizes an *h*_Vessel_/*D*_Vessel_ of 3/1 with a working volume of 50% (*h*_L_/*D*_Vessel_ = 1.5) was assumed, leading to Equation (2) for the height of the liquid *h*_L, sim_.

(1) $$p_{\mathrm{hydro}}=h_{L}*g*\rho =\left(\frac{\pi *D^{2}}{4*V_{L}}\right)*g*\rho$$

(2) $$h_{L,\mathrm{sim}}=\sqrt[{3}]{\frac{9\;V_{L}}{\pi }}$$

A plane was fitted to the data generated during the dissolved CO_2_ assessment (2.2) using the OriginPro software to generate an Equation (3), presented in the result section, to assess the influence of Δ*p*_Vessel_ and Δ*p*_Hydro_ on the dissolved CO_2_ concentration. This Equation (3) was further used to estimate the dissolved CO_2_ concentrations in different reactor sizes and pressure conditions. The maximum working volume was inferred for all three experimental conditions in which the effect of Δ*p*_Vessel,_ on the dissolved CO_2_ concentration is compensated fully with the increase in Δ*p*_Hydro_.

All error bars and uncertainties presented are calculated as standard error or as the result of the Gaussian error propagation law of the former. Statistical differences were evaluated with a two‐tailed Student's t‐test at a level of significance (α) of 5%, 1%, and 0.1% under the assumptions of normally distributed samples with equal variances.

## 3 Results and Discussion

### 3.1 Effect of Pressure on CO_2_ Saturation Values

**FIGURE 2:**
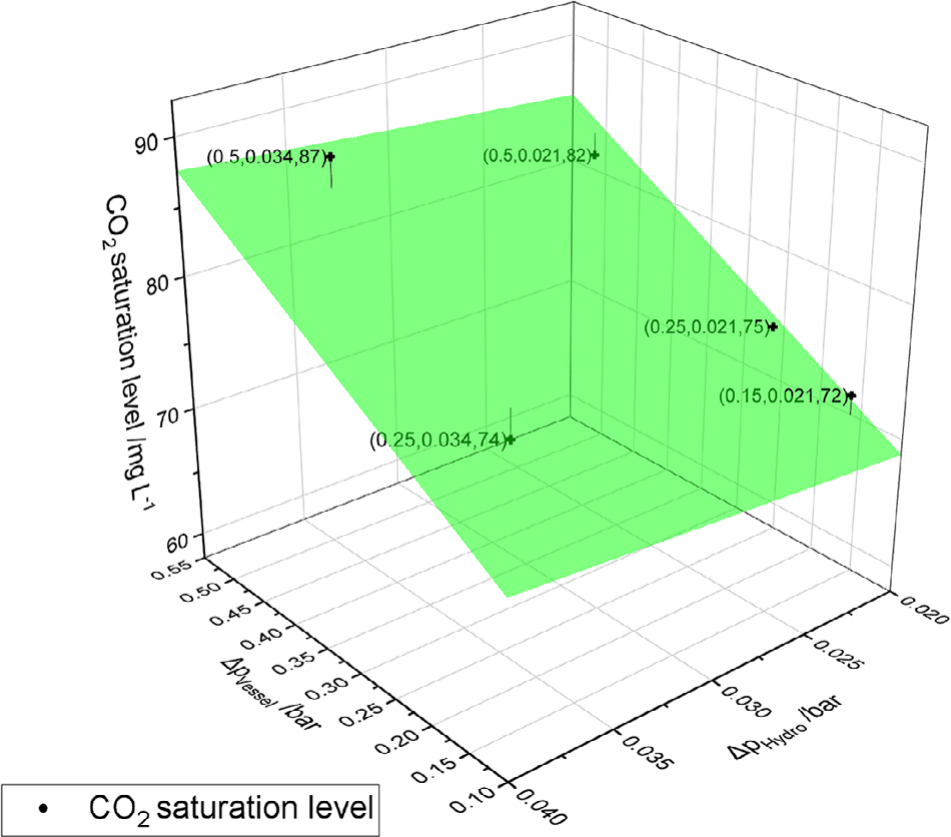
Mapping of the CO_2_‐saturation dependency of applied and hydrostatic pressure.

The CO_2_ saturation values were evaluated in the technical scale. Five process conditions were systematically tested, and the CO_2_ saturation levels were analyzed. Figure 2 illustrates the map of CO_2_ saturation with respect to applied pressure (Δ*p*_Vessel_) of the fermenter (between 0.15–0.55 bar) and hydrostatic pressure (Δ*p*_Hydro_). A plane was fitted to the data, which described it with an R^2^ of 0.91. The corresponding Equation (3) shows a clear influence of the applied pressure on the CO_2_ saturation with 36 mg L^−1^ bar^−1^. The hydrostatic pressure also exhibits an influence with 117 mg L^−1^ bar^−1^, which shows that increased hydrostatic pressures in large‐scale reactors could be significant and should be taken into consideration. The offset of the equation can be attributed to the saturation of CO_2_ due to the atmospheric pressure, since only the additional applied pressure has been taken into account. It can be concluded that by altering Δ*p*_Vessel_ and Δ*p*_Hyydro_, the solubilities of the gases can be altered. The gathered data is above previously reported concentrations for autotrophic growth (1–8 mg L^−1^) for *C. necator sp*. with CO_2_ as carbon source [23], which shows that the $C_{{\mathrm{CO}}_{2}}$ is sufficient to be used by the organism.

(3) $$\begin{array}{ccc} c_{CO_{2}}\left[\mathrm{mg}\;L^{-1}\right] & = & z_{0}+a*\Delta p_{\mathrm{Vessel}}+b*\Delta \;p_{\mathrm{Hydro}}\\ & = & 63\;\mathrm{mg}\;L^{-1}+36\;\frac{\mathrm{mg}\;L^{-1}}{\mathrm{bar}}*\Delta p_{\mathrm{Vessel}}\\ & & +\;117\;\frac{\mathrm{mg}\;L^{-1}}{\mathrm{bar}}\Delta p_{\mathrm{Hydro}} \end{array}$$

### 3.2 Growth Performance

The effect of Δ*p*_Vessel_ and Δ*p*_Hydro_ on the bacterial growth and on the polymer production was evaluated in three separate fermentation setups. Comparative analysis of the technical‐scale fermentation at decreased pressure (DP), the technical scale fermentation with increased pressure (IP), and the pilot scale fermentation with increased pressure (pilot) was used to determine whether observed changes could be attributed solely to Δ*p*_Vessel_ or whether Δ*p*_Hydro_ had a significant impact as well.

All fermentations were initiated with a biomass growth phase on glucose. Once glucose was depleted, LA was introduced by the pH–pO_2_‐dependent feeding. Here, residual nitrogen was measured between 0.8–1.0 g_NH4+_ L^−1^ and provided the base for cell growth on LA. Onset of PHA production was observed once c_NH4+_ was at 0.1 g_NH4+_ L^−1^.

**FIGURE 3:**
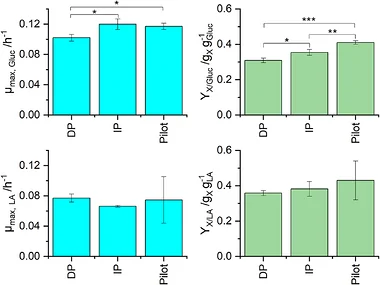
Growth parameters on glucose and levulinic acid as carbon substrate with the maximum specific growth rates µ_max_ and the biomass yields Y_X/S_ ; “DP” and “IP” referring to fermentations with 23 L initial V_L_ and “Pilot” with 530 L initial V_L_; significant differences between two values of the same metric are labeled with * (5%), ** (1%), and *** (0.1%) for significance level.

An overview of the key growth parameters during both growth phases in the different fermentations is provided in Figure 3.

During the growth phase on glucose, a positive influence is seen with an increase in Δ*p*_Vessel_. Both fermentation with increased Δ*p*_Vessel_ (IP & Pilot) show a significantly (DP–IP: α = 5%; DP–Pilot: α = 5%) increased specific growth rate with *µ*_max_ of 0.120 ± 0.007 h^−^^1^ and 0.117 ± 0.004 h^−^^1^ compared to fermentation at decreased pressure with 0.102 ± 0.004 h^−^^1^. This advantageous influence of applied pressure can be linked to foam formation: In the DP fermentation, foaming occurred during the growth phase on glucose with seven triggers of the level probe (antifoam) between 17–37 h. This foaming was effectively prevented by increased Δ*p*_Vessel_ of the reactor during the IP fermentation, with no signal during the growth phase on glucose in the IP fermentation in the technical scale and only one occurrence at 25 h in the pilot scale. Every time foam forms, it dissipates cell mass, which remains on the reactor walls and is no longer available for further division. Furthermore, addition of antifoam agent might also decrease the cell viability. This reduces the overall growth rate and explains significant reduction in the technical DP fermentation, which repeatedly lost active biomass.

The biomass yield on glucose (Y_X/Gluc_) showed a dependency not only on Δ*p*_Vessel_, but also increased with Δ*p*_Hydro_ (DP–Pilot: α = 0.1%; IP–Pilot: α = 1%) from 0.31 ± 0.01 g_X_ g_Gluc_^−^^1^ to 0.35 ± 0.02 g_X_ g_Gluc_^−^^1^ and 0.41 ± 0.01 g_X_ g_Gluc_^−^^1^. The increase was expected, due to the reassimilation of autogenously produced CO_2_ as already described by Shimizu et al. [10].

### 3.3 Influence of Fermentation Pressure on the 3HV Content

**FIGURE 4:**
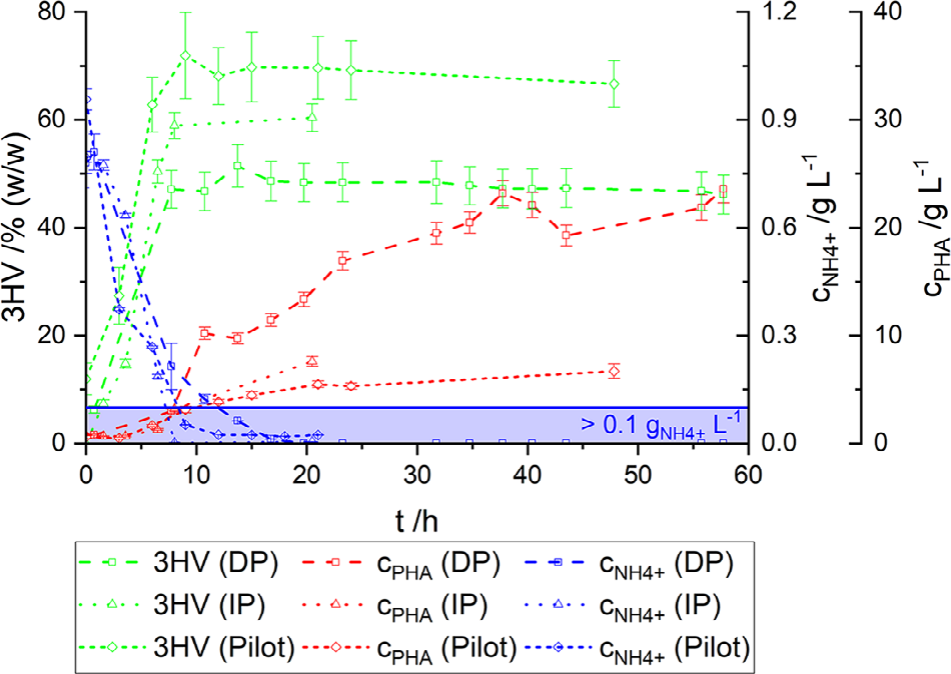
Concentration of PHA c_PHA_, ammonia c_NH4+_ and the 3HV‐content with decreased pressure (□), increased pressure (△), and in the pilot scale (◇), respectively with levulinic acid as carbon substrate; “DP” and “IP” referring to fermentations with 23 L initial V_L_ and “Pilot” with 530 L initial V_L_; *t* = 0 as the first sample with measured LA, the complete fermentation profiles are depicted in appendix 2.

Once LA was introduced as a carbon substrate, the 3HV content of the polymer began to increase. Figure 4 compares the time course of this phase for all three process conditions. It can be observed that minor quantities of PHA are already present while nitrogen is still present. However, only once residual nitrogen in the medium is taken up (c_NH4+_ <0.1 g L^−1^), a noticeable increase in PHA production is visible and the 3HV content stabilizes. In all three fermentations, the concentration of PHA increases, while the composition stays constant. This shows that the ratio of the produced monomers remains unchanged during the production phase, which allows us to take all the values from the production phase into consideration when investigating the underlying mechanism.

**FIGURE 5:**
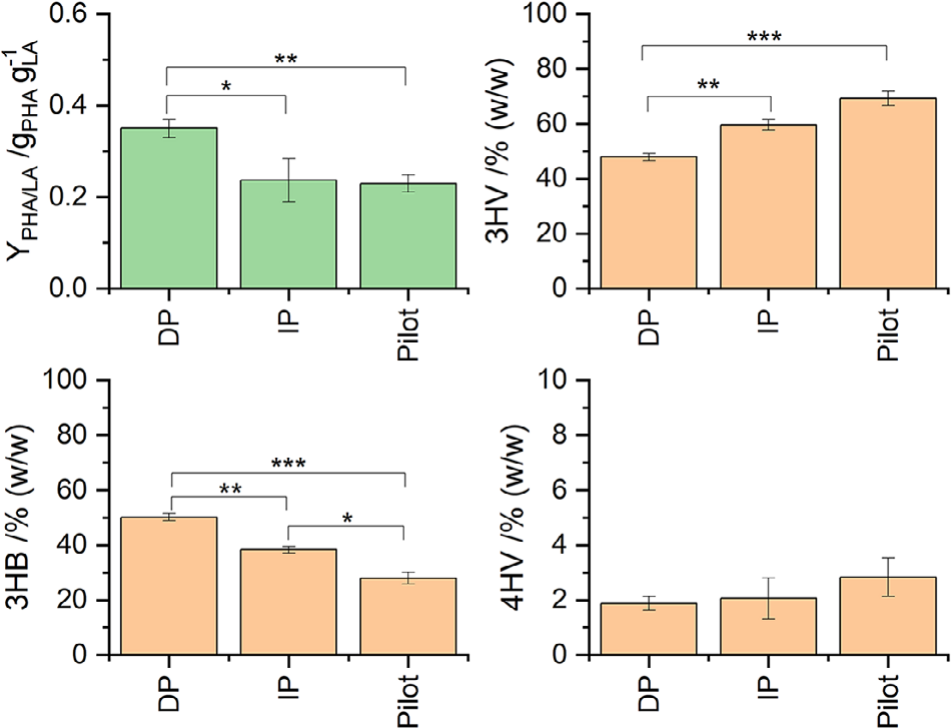
Production parameters with average monomer fractions during the production phase and the product yields Y_PHA/LA_ ; “DP” and “IP” referring to fermentations with 23 L initial V_L_ and “Pilot” with 530 L initial V_L_; significant differences between two values of the same metric are labeled with * (5%), ** (1%), and *** (0.1%) for significance level.

Comparing the production parameters depicted in Figure 5 a significant decrease in polymer yields Y_PHA/LA_ and a shift in the monomer fractions of the produced polymer can be observed with changing fermentation pressures. Statistical analysis confirmed a strongly significant decrease in Y_PHA/LA_ (DP – IP: α = 5%, DP – Pilot: α = 1%), which is further discussed in chapter 3.4 and an increase in the mean 3HV content with rising pressure (DP – IP: α = 1%, DP – Pilot: α = 0.1%). As expected, 3HB content behaved in contrary to the 3HV content (DP – IP: α = 1%, DP – Pilot: α = 0.1%, IP – Pilot: α = 5%). The 4HV content does not seem to be significantly impacted by changing process conditions, which is expected, since its formation is not dependent on the balance of acetyl‐CoA and propionyl‐CoA (Figure 1).

**FIGURE 6:**
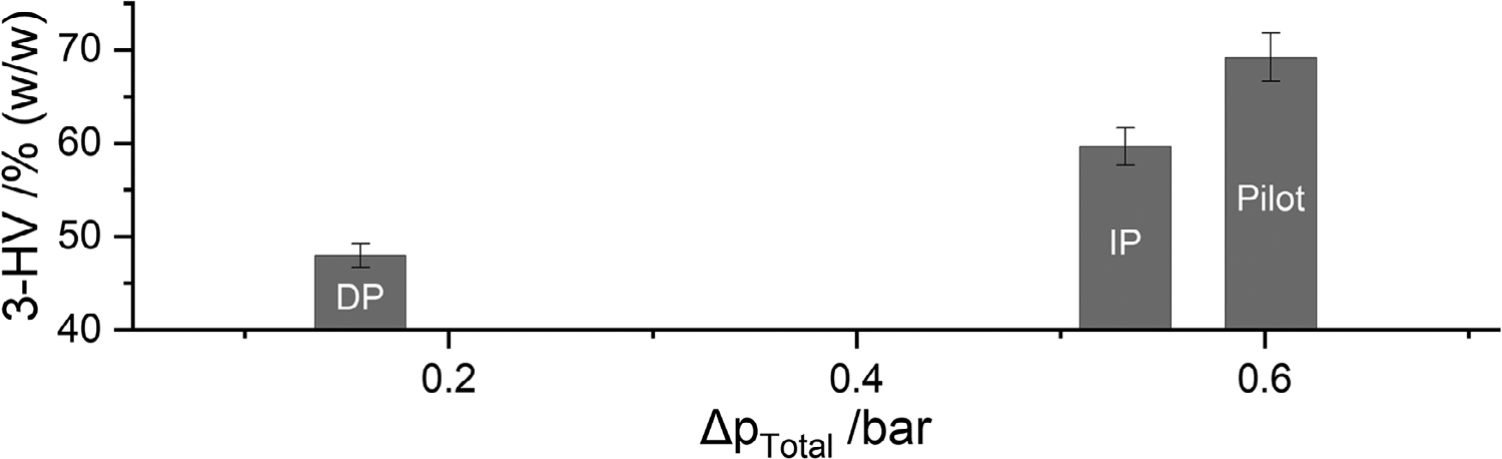
Average 3HV content as a function of Δp_Total_ (Δp_Vessel_ + Δp_Hydro_) during the production phase with “DP” and “IP” referring to fermentations with 23 L initial V_L_ and “pilot” with 530 L initial V_L._

To further investigate the dependency of the monomer fraction on the dissolved CO_2_, Figure 6 links the shift of the 3HV content to the rising total additional pressure as obtained as the sum of Δ*p*_Vessel_ and Δ*p*_Hydro_. As expected, with increasing fermenter pressure, an increase in 3HV content was observed. This may be directly linked to enhanced CO_2_ solubility in the fermentation broth at elevated pressures. If increased pressure enhances the availability of dissolved CO_2_, reassimilation of autogenously CO_2_ via the CBB cycle is likely to be promoted. Consequently, elevated dissolved CO_2_ concentrations may increase the demand for ATP and reducing equivalents required for operation of the CBB cycle. To satisfy this demand, a larger fraction of acetyl‐CoA may be directed toward energy‐generating pathways, such as the TCA cycle instead of polymer biosynthesis. Since acetyl‐CoA is the direct precursor for 3HB formation, while 3HV formation additionally depends on propionyl‐CoA, a reduction in the relative availability of acetyl‐CoA could increase the intracellular propionyl‐CoA/acetyl‐CoA ratio. Such a shift would be consistent with the observed increase in 3HV incorporation and the simultaneous reduction in PHBVV yield. The simultaneous increase in 3HV content and decrease in PHBVV yield suggests that pressure‐induced increases in dissolved CO_2_ concentrations may affect not only monomer distribution, but also the overall allocation of carbon within the cell.

Overall, these results suggest that elevated CO_2_ concentrations resulting from increased pressure can induce a metabolic shift toward higher HV incorporation. This pressure‐dependent modulation of monomer composition provides a promising strategy for tailoring PHBVV properties by adjusting applied and hydrostatic pressure during fermentation.

**FIGURE 7:**
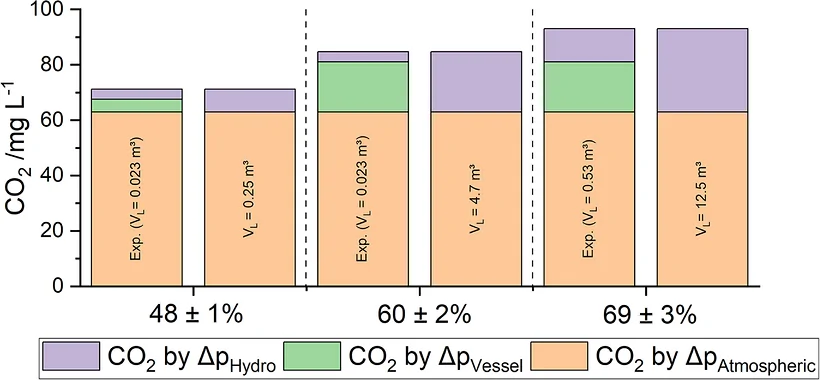
Estimated dissolved $c_{{\mathrm{CO}}_{2}}$ = f(Δp_Vessel_ + Δp_Hydro_) for different working volumes and pressure conditions using Equation (3), bars are grouped by the corresponding experimental 3HV contents.

Using Equation (3), estimated values for $c_{{\mathrm{CO}}_{2}}$ and the influence of Δ*p*_Vessel_ and Δ*p*_Hydro_ can be obtained (Figure 7). This allows us to predict the largest fermenter volume, in which the specific polymer composition can be produced. For example, to reach a 3HV content of 48% in the experimental setup we had a liquid volume of 0.023 m^3^ corresponding to an added $3.7\;{\mathrm{mg}}_{{\mathrm{CO}}_{2}}$ L^−1^ by Δ*p*_Hydro_. Together with the influence of the Δ*p*_Vessel_ ($4.5\;{\mathrm{mg}}_{{\mathrm{CO}}_{2}}$ L^−1^) and the offset by atmospheric pressure ($63.0\;{\mathrm{mg}}_{{\mathrm{CO}}_{2}}$ L^−1^) we reach a total concentration of $71.2\;{\mathrm{mg}}_{{\mathrm{CO}}_{2}}$ L^−1^. If we want to scale up the process for this specific 3HV content to 0.25 m^3^ the elevated Δ*p*_Hydro_ adds $8.2\;{\mathrm{mg}}_{{\mathrm{CO}}_{2}}$ L^−1^ totaling to $71.2\;{\mathrm{mg}}_{{\mathrm{CO}}_{2}}$ L^−1^ without any Δ*p*_Vessel_.

According to Equation (3), this additional contribution of Δ*p*_Hydro_ compensates for the contribution of Δ*p*_Vessel_ applied in the smaller reactor, which should result in a comparable dissolved CO_2_ concentration and therefore a comparable amount of 3HV content.

An additional increase in volume would need additional measures to reduce the CO_2_ in the broth to keep the concentration stable and produce comparable polymer compositions. Consequently, in smaller volumes, Δ*p*_Vessel_ needs to be increased to compensate for lower Δ*p*_Hydro_. Similar assessments for all three experimental conditions are presented in Figure 7 and should be considered when scaling up PHBVV production process.

It should be noted that Equation (3) should be regarded as an empirical engineering approximation. In larger industrial reactors, additional factors such as gas hold‐up, mixing heterogeneity, and CO_2_ stripping efficiency may influence the actual dissolved CO_2_ concentration. Consequently, the model is intended to illustrate general trends and support scale‐up considerations within the investigated operating range rather than to provide a universal prediction for all reactor scales.

While pressure‐dependent control of polymer composition may provide a useful tool for tailoring PHBVV properties, practical implementation at industrial scale requires consideration of economic and technical constraints. Increased operating pressure requires more robust equipment and increases safety requirements. However, hydrostatic pressure naturally increases with reactor size and therefore influences dissolved CO_2_ concentrations even without intentional vessel pressurization. Consequently, pressure effects should be considered during process scale‐up regardless of whether pressure is actively used as a process control parameter.

### 3.4 Potential Toxicity of High HV Contents on Cells

While the modulation of the 3HV content via Δ*p*_Total_ is promising, overall production efficiency must also be taken into consideration. The product yields (Figure 5) are significantly reduced in the IP fermentation and the pilot scale (0.23 ± 0.02 g_PHA_ g_LA_^−^^1^, 0.24 ± 0.05 g_PHA_ g_LA_^−^^1^; DP–Pilot: α = 1%; DP–IP: α = 5%) compared to the DP fermentation (0.35 ± 0.01 g_PHA_ g_LA_^−^^1^). Besides the proposed metabolic burden by increased CO_2_ fixation, additional factors may contribute to the reduced PHA yield at elevated pressure. Elevated dissolved CO_2_ concentrations may alter intracellular pH homeostasis by their influence on the carbonic acid buffer, increase maintenance requirements, and affect membrane‐associated processes. While these aspects were not directly investigated in this study, they may contribute to the observed reduction in polymer productivity. These factors should be considered in future studies.

One factor in addition to the metabolic burden is inferred from the severe foaming or cell lysis in both fermentation setups with 0.5 bar *p*_Vessel_ and increased 3HV content, which led to the discontinuation of the experiments. This phenomenon suggests a possible association between increased 3HV incorporation and cellular stress.

Support for this hypothesis is provided by differences in the physicochemical properties of the monomer precursors. Leonhardt et al. [24] linked the partition coefficients (logP) of butyrate and valerate to their inhibitory concentration (IC_50_) in *C. necator* H16. They showed that an increase of the logP from 0.8 (butyric acid) to 1.4 (valeric acid) reduces the IC_50_ of the respective acids from 5.2 to 3.9 g L^−1^, which shows that higher logP can be linked to greater inhibitory character. While Leonhardt et al. [24] evaluated the IC_50_ based on the extracellular acid concentration, the metabolization of the substrates happens intracellular in the cytoplasm. Due to the so‐called PHA‐cycle, the simultaneous production and degradation of the PHA granules, the intermediates of PHBVV synthesis are also always present in the cytoplasm [25]. Using the tool SwissADME [26], the logP of the intermediates was estimated as −0.19 for monomeric 3HB_aq_ and 0.12 for 3HV_aq_, indicating a higher inhibition by 3HV_aq_, which is consistent with the trend presented by Leonhardt et al. [24] for butyric and valeric acid.

Obruca et al. [25] showed that the dissolved intracellular 3HB_aq_ concentration in *C. necator* can be as high as 16.5‐fold the level of a non‐producing mutant [25] and that 3HB_aq_ can stabilize proteins serving as a chaperone. Assuming similar levels of 3HV_aq_, they may destabilize the cell by affecting the lipid bilayers due to the higher hydrophobicity of 3HV_aq_, potentially compromising membrane integrity. Combined with the missing stabilizing effect of high 3HB_aq_ levels, such destabilization could explain the observed cell lysis and excessive foaming under conditions favoring high 3HV incorporation.

Taken together, these findings suggest that while pressure‐induced tuning of PHBVV composition is feasible, elevated 3‐hydroxyvalerate fractions may impose physiological limits on the cells.

However, this conclusion remains conjectural and requires dedicated physiological investigations to assess the role of cellular stress and toxicity associated with high 3HV contents to ensure robust and scalable fermentation performance.

## 4 Conclusion

Increased applied and hydrostatic pressures, resulting in higher CO_2_ solubilities, were shown to significantly influence production‐related parameters, including PHA yield and polymer composition. These effects may be linked to enhanced CO_2_ fixation with higher levels of dissolved CO_2_.

Scaling the process to pilot scale provided critical insights into the role of hydrostatic pressure on substrate utilization and biomass formation, revealing that increased pressure can enhance growth performance, while simultaneously affecting metabolic fluxes toward polymer synthesis. Importantly, the pilot‐scale experiments highlighted potential physiological limits, such as reduced PHA yields and process instability, underscoring the need to balance productivity with cellular robustness during scale‐up.

Overall, the findings propose fermenter pressure as a key, yet often overlooked, process parameter for controlling polymer composition and process performance. The knowledge gained in this study provides a solid basis for the rational scale‐up of PHBVV production toward industrial scale, enabling the maintenance of cell vitality and targeted polymer properties, while accounting for pressure‐dependent metabolic effects.

## Author Contributions

**Birk Achenbach**: Conceptualization, methodology, investigation, formal analysis, visualization, writing – original draft, and writing – review and editing. **Pravesh Tamang**: Conceptualization, project administration, supervision, writing – original draft, writing – review and editing, and visualization. **Günter E. M. Tovar**: Writing – review and editing and supervision. **Susanne Zibek**: Conceptualization, project administration, funding acquisition, supervision, visualization, writing – review and editing, and resources.

## Conflicts of Interest

The authors declare no conflicts of interest.

## Supporting information




**Supporting File 1**: biot70298‐sup‐0001‐Appendix1.docx.

**Supporting File 2**: biot70298‐sup‐0002‐Appendix2.docx.

## Data Availability

The data that support the findings of this study and are not already presented in the manuscript are available on request from the corresponding author.
