# Supplementary material for: Pressure Dependent Changes in CO2‐Concentrations Modulate the 3‐Hydroxyvalerate Fraction in Terpolymeric Polyhydroxyalkanoates
Source: Biotechnol J. 2026 Aug 20;21(8):e70298. doi: 10.1002/biot.70298 (PMC13490969; doi:10.1002/biot.70298)
Supplement: Supplementary file 1 [file BIOT-21-e70298-s001.docx]

| Parameter | Technical DP | Technical IP | Pilot |
| --- | --- | --- | --- |
| Growth Parameter | | | |
| µ_max, Gluc_ /h^-1^  (Δt) | 0.102 ± 0.004  (1.5 – 37.0 h) | 0.120 ± 0.007  (3.0 – 25.8 h) | 0.117 ± 0.004  (0.0 – 28.8 h) |
| µ_max, LA_ /h^-1^  (Δt) | 0.077 ± 0.005  (38.0 – 48.0 h) | 0.066 ± 0.001  (28.3 – 34.9 h) | 0.08 ± 0.03  (30.0 – 36.0 h) |
| Y_x/Gluc_ /g_X_ g_Gluc_^-1^ | 0.31 ± 0.01 | 0.35 ± 0.02 | 0.41 ± 0.01 |
| Y_x/LA_ /g_X_ g_LA_^-1^ | 0.36 ± 0.02 | 0.38 ± 0.04 | 0.4 ± 0.1 |
| Production Parameter | | | |
| c_x_ / g L^-1^ | 31.2 ± 1.0 | 12.5 ± 0.5 | 19.6 ± 0.5 |
| PHA / %_CDW_ | 76 ± 3 | 61 ± 3 | 34 ± 3 |
| Y_PHA_ /g_PHA_ g_LA_^-1^ | 0.35 ± 0.02 | 0.24 ± 0.05 | 0.23 ± 0.02 |
| 3-HB / %_(w/w)_^a^ | 50 ± 1 | 38 ± 1 | 28 ± 2 |
| 3-HV / %_(w/w)_^a^ | 48 ± 1 | 60 ± 2 | 69 ± 3 |
| 4-HV / %_(w/w)_^a^ | 1.9 ± 0.2 | 2.1 ± 0.7 | 2.8 ± 0.7 |
| ^a^ Mean during production phase with c_NH4+_ < 0.1 g L^-1^ | | | |

Appendix 1: Fermentation results of different parameter variations
