# Supplementary material for: Pressure Dependent Changes in CO2‐Concentrations Modulate the 3‐Hydroxyvalerate Fraction in Terpolymeric Polyhydroxyalkanoates
Source: Biotechnol J. 2026 Aug 20;21(8):e70298. doi: 10.1002/biot.70298 (PMC13490969; doi:10.1002/biot.70298)
Supplement: Supplementary file 2 [file BIOT-21-e70298-s002.docx]

Appendix 2 Fermentation profiles
